# Exploring the role of exosomal MicroRNAs as potential biomarkers in preeclampsia

**DOI:** 10.3389/fimmu.2024.1385950

**Published:** 2024-03-19

**Authors:** Yuping Shan, Bo Hou, Jingli Wang, Aiping Chen, Shiguo Liu

**Affiliations:** ^1^ Department of Obstetrics and Gynecology, The Affiliated Hospital of Qingdao University, Qingdao, China; ^2^ Department of Cardiovascular Medicine, The Affiliated Hospital of Qingdao University, Qingdao, China; ^3^ Department of Medical Genetics, The Affiliated Hospital of Qingdao University, Qingdao, China

**Keywords:** biomarkers, epigenomics, exosomes, microRNAs, preeclampsia

## Abstract

The complex pathogenesis of preeclampsia (PE), a significant contributor to maternal and neonatal mortality globally, is poorly understood despite substantial research. This review explores the involvement of exosomal microRNAs (exomiRs) in PE, focusing on their impact on the protein kinase B (AKT)/hypoxia-inducible factor 1-α (HIF1α)/vascular endothelial growth factor (VEGF) signaling pathway as well as endothelial cell proliferation and migration. Specifically, this article amalgamates existing evidence to reveal the pivotal role of exomiRs in regulating mesenchymal stem cell and trophoblast function, placental angiogenesis, the renin–angiotensin system, and nitric oxide production, which may contribute to PE etiology. This review emphasizes the limited knowledge regarding the role of exomiRs in PE while underscoring the potential of exomiRs as non-invasive biomarkers for PE diagnosis, prediction, and treatment. Further, it provides valuable insights into the mechanisms of PE, highlighting exomiRs as key players with clinical implications, warranting further exploration to enhance the current understanding and the development of novel therapeutic interventions.

## 1 Introduction

Preeclampsia (PE) is a syndrome characterized by hypertension and concurrent multisystemic dysfunction during pregnancy (1). It affects approximately 2–8% of pregnancies worldwide, and the incidence of PE varies among different countries and regions, with estimates ranging from at least 16% in low-income and middle-income countries to more than 25% in certain countries in Latin America (2). Globally, preeclampsia causes the loss of 76,000 pregnant women and 500,000 fetuses every year, seriously endangering the health of pregnant women and perinatal infants (3). Furthermore, previous exposure to PE is projected to increase the risk of chronic health problems for around 300 million women and children worldwide (4). This places a significant burden on families and society, making it an important global public health issue (5). According to the International Society for the Study of Hypertension in Pregnancy (ISSHP), PE can be defined as a progressive disease of pregnancy involving multiple organ systems. PE can be diagnosed after 20 weeks of gestation by new-onset hypertension (systolic blood pressure ≥140 mmHg and/or diastolic blood pressure ≥90 mmHg; average of two measurements) in a patient previously with normotension plus one other pre-eclampsia-related symptom or sign. These can include proteinuria (protein/creatinine ratio ≥30 mg/mmol in a spot urine sample or ≥300 mg/mmol in >0.3 g/day), acute kidney injury (creatinine ≥90 µmol/l), liver involvement (elevated transaminases), neurological symptoms (eclampsia, altered mental status, blindness, stroke, clonus, severe headaches, persistent visual scotomata), hematological abnormalities (thrombocytopenia, disseminated intravascular coagulation, hemolysis), cardiorespiratory complications (pulmonary edema, myocardial ischemia or infarction, oxygen saturation <90%, ≥0%, inspired oxygen for more than 1 hour, intubation other than for cesarean), or uteroplacental dysfunction (placental abruption) (4). The condition poses significant risks to maternal health, including potentially fatal outcomes such as disseminated intravascular coagulation, eclampsia, cerebral hemorrhage, pulmonary edema, acute renal damage, hepatic failure, and stroke, making PE the second leading cause of maternal death in clinics (6). A strong correlation has been reported between a history of PE and future cardiovascular or cerebrovascular disease risk, emphasizing the long-term implications of this condition in the mother (7). Moreover, PE contributes to increased fetal morbidity and mortality through iatrogenic preterm delivery, fetal growth restriction, and placental abruption (8).

**Figure 1:**
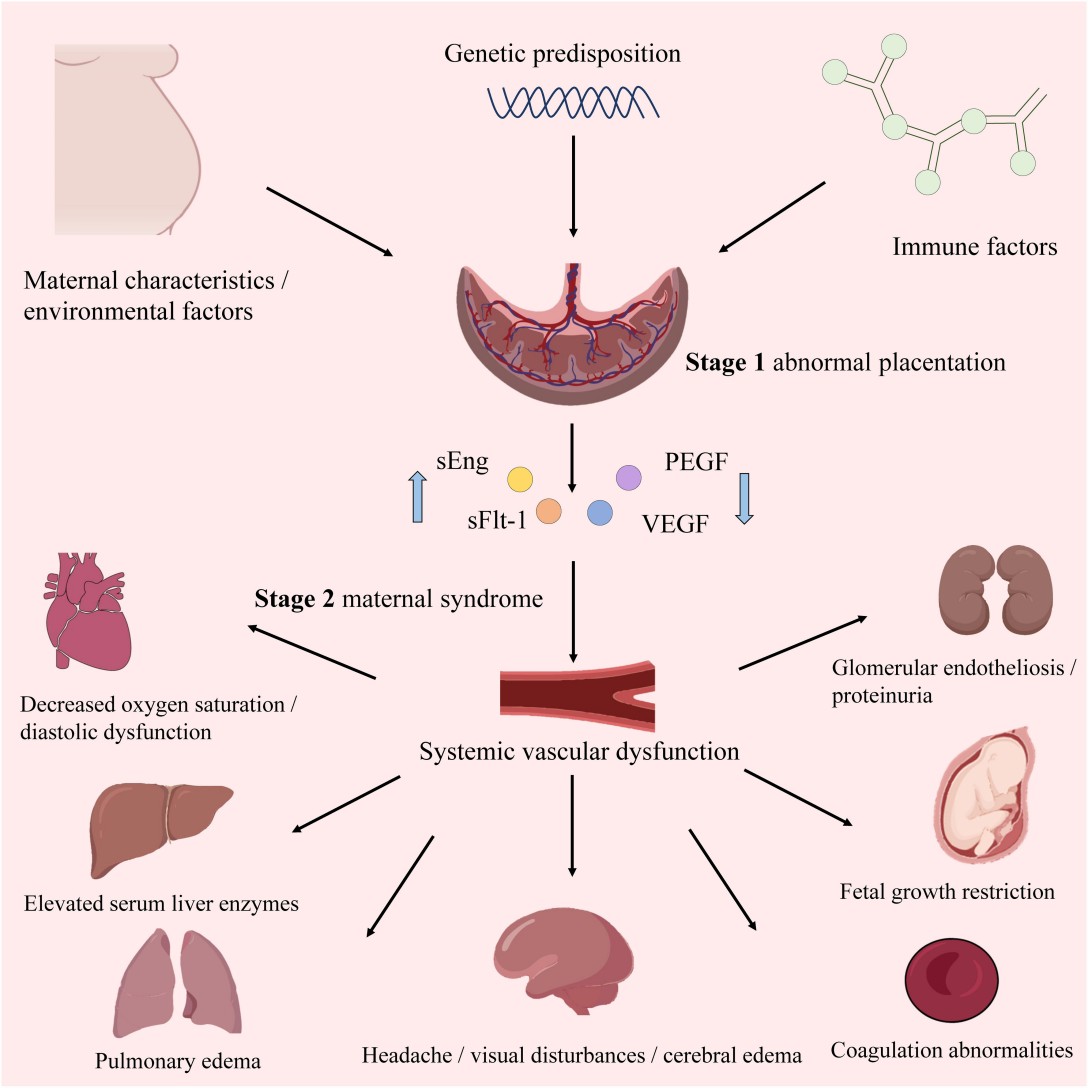
The overview of PE. The upward blue arrow represents “increase”, and the downward blue arrow represents “decrease”.

Similar to ISSHP guidelines, the American College of Obstetricians and Gynecologists (ACOG) and the National Institute for Health and Care Excellence (NICE) guidelines recognize that high-risk factors include obstetric history and maternal factors (4). Moreover, NICE and ACOG have released risk assessment guidelines based on maternal characteristics and medical history. The pathogenesis of PE is multifactorial, involving two stages. The first is abnormal placentation in early pregnancy due to insufficient extravillous trophoblast (EVT) invasion and suboptimal spiral artery remodeling. The second stage comprises placental ischemia/reperfusion injury leading to maternal angiogenic imbalance, immune-mediated response, and endothelial cell dysfunction (9) (**Figure 1**). Despite advancements, the specific mechanisms underlying PE pathogenesis and etiology remain elusive. In particular, the accumulation of reactive oxygen species (ROS) resulting from heightened oxidative stress (OS) plays a significant role in the two stages, making it a key indicator of PE (10). Additionally, impaired spiral artery remodeling, poor placental implantation, and abnormal levels of angiogenic proteins in maternal blood are also important indicators of PE (11). The ISSHP guidelines recommend oral aspirin, low dietary calcium intake, and exercise to reduce the likelihood of PE (12). Once diagnosed, the management method involves delivering the baby and placenta at full term gestation, or, if preterm PE is detected, expectant management can be used until a more advanced gestation is reached (11).

Current consensus suggests that PE arises from a combination of factors, including aberrant placental function, immune-system modifications, inflammatory activity, imbalanced angiogenic and anti-angiogenic factors, and metabolic abnormalities (11). Additionally, genetic variation, trace elements, changes in lipid metabolism, and OS in pregnant women have been implicated in PE pathophysiology (13). Emerging evidence points to the role of epigenetics, particularly microRNAs (miRNAs), in PE pathogenesis (14). For example, certain miRNAs (e.g., miR-182 and miR-210) are dysregulated in individuals with PE compared to normal pregnancy (15). Notably, exosomal microRNAs (exomiRs) have also been implicated in PE, including miR-153-3p and miR-517a (8). Tkachenko et al. assumed that the altered level of miR-210 could influence the expression of specific miRNAs in the development of PE, including miR-1, miR-27a, miR-29a, miR-130a, miR-152, miR-193b, and miR-519b (16). Escudero et al. proposed that the synthesis of exosomes containing miRNAs is influenced by various factors such as hypoxia and the balance between pro-oxidative and anti-oxidative mechanisms, and they may contribute to the significant changes in endothelial protein expression observed in PE, resulting in endothelial dysfunction in both maternal and maternal-fetal circulation and subsequent impairment of angiogenesis, which is the key feature of PE (1).

Owing to gaps in knowledge and limitations in early detection methods, effective preventive and treatment options for PE remain lacking (17). Current approaches for predicting PE occurrence lack sufficient sensitivity. Meanwhile, exosomes, known to transport miRNAs to distant organs, may play a crucial role in the systemic organ damage associated with PE and may serve as significant predictors of the condition (18). However, despite some studies reporting the potential endothelial regulatory role of exomiRs, they have not proven to be reliable predictors for PE, and their application poses a significant challenge (1). This review provides a comprehensive overview of the current evidence on exomiR function in PE and explores the potential utility of exomiRs in diagnosis, prediction, and treatment. By combining existing knowledge, this review enhances the understanding of PE pathogenesis and may contribute to the development of novel management strategies. We hope that this review will encourage more researchers to pay attention to the role of exomiRs as biomarkers, thereby contributing to the diagnosis and treatment of PE.

## 2 PE epigenetics

**Figure 2:**
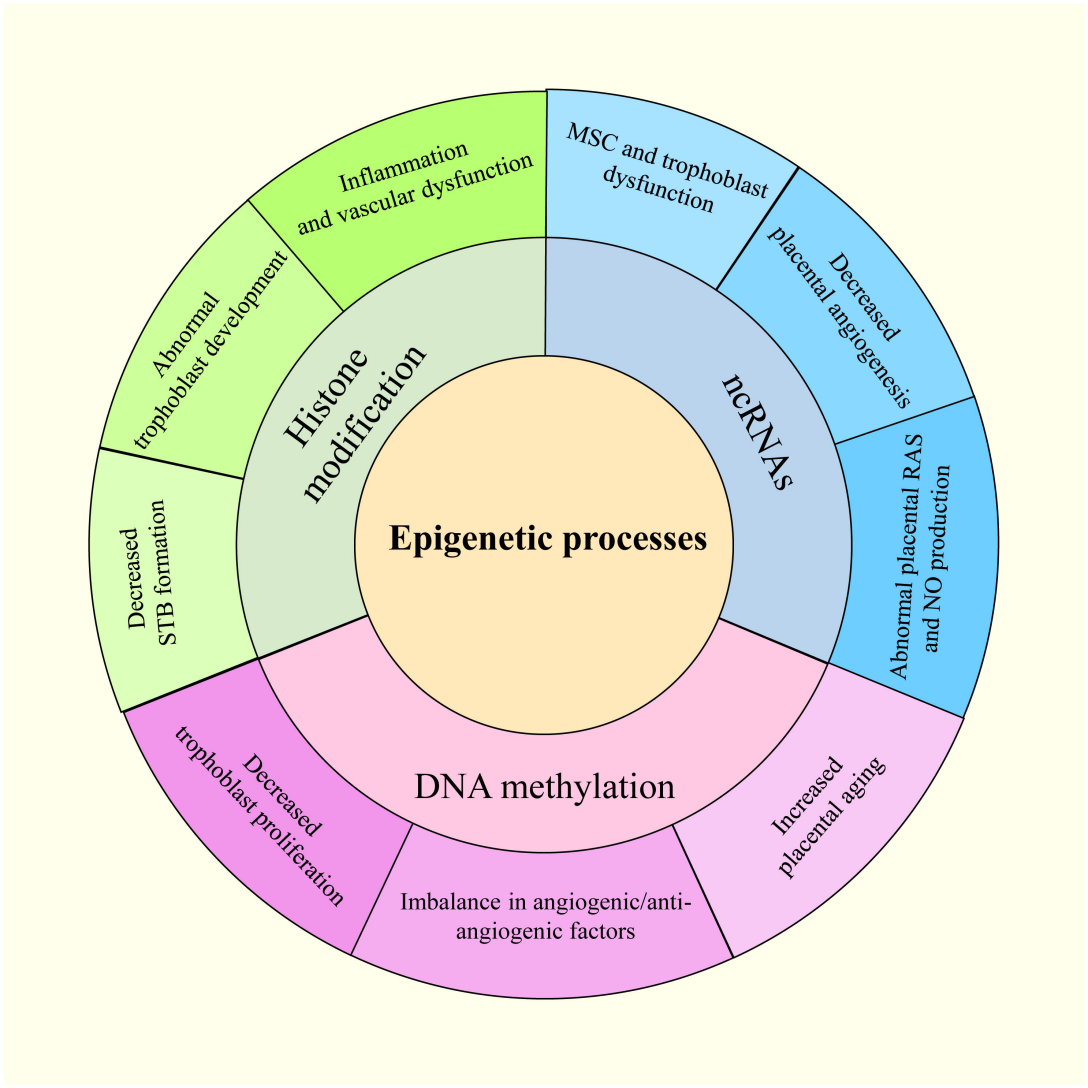
The epigenetic modifications in PE.

Waddington first used the term “epigenetics” in the 1940s to describe how an organism’s environment and genes might interact to cause non-Mendelian inheritance of phenotypes (19). This definition has evolved to “molecular factors and processes around DNA that regulate genome activity, independent of DNA sequence, and are mitotically stable” (20). Epigenetic modifications result from environmental changes that affect biological processes within an organism, affecting heritable variations in gene expression (21). The epigenetic disruption of gene expression patterns can lead to autoimmune disorders, cancers, and other diseases (22). DNA methylation, histone modifications, and non-coding RNAs (ncRNAs) are three basic epigenetic codes that have received considerable attention (23). Histone and DNA modifications play intermediate roles in controlling gene activity (24). Moreover, altering functional ncRNA regulation can modify gene activity, affecting chromatin structure, epigenetic memory, selective RNA splicing, and protein translation (24). Notably, gestational hypoxia permits adaptive reactions to modifications in the placental environment in preterm infants through epigenetics (25). In addition, PE and other pregnancy-related complications may be influenced by miRNAs belonging to ncRNAs (26). The overview of the epigenetic process in PE is shown in **Figure 2**.

### 2.1 DNA methylation during PE

DNA methylation, the most common epigenetic mechanism, involves the addition of a methyl group to cytosine-phospho-guanine (CpG) dinucleotide sites, catalyzed by DNA methyltransferases (DNMTs) (27). CpG sites are frequently located in genomic regions known as CpG islands, comprising nearly half of the human genome, and are frequently targeted by transcription factors (28). DNMT1 is responsible for maintaining genome-wide methylation, whereas DNMT3A and DNMT3B initiate *de novo* DNA methylation, establishing new methylation patterns (29). DNA methylation, serving as heritable epigenetic markers, is crucial to embryonic development, transcription, chromatin structure, and X-chromosome inactivation and can repress transcription or post-transcriptional RNA degradation to silence downstream target genes (25). Alterations in gene expression in the placentas of women with PE have been potentially attributed to epigenetic modifications induced by the abnormal placental environment (30). Further, most studies showing alterations in DNA methylation related to placental development gene expression in PE have reported direct correlations (31). Mayne et al. discovered 62 sites of abnormal DNA methylation in early PE linked to increased placental aging (32). Furthermore, Zadora et al. observed upregulation of the homeobox gene family, exhibiting diverse methylation patterns, including *TLX1* and *DLX5*, which correlated with decreased trophoblast proliferation and PE (33). Additionally, Wang et al. reported elevated expression of vascular endothelial growth factor A (VEGFA), VEGFC, hypoxia-inducible factor 1-α (HIF1α), and *RELA* proto-oncogene associated with attenuation of the CpG island methylator phenotype (34). These findings suggest associations between PE and dysregulated angiogenic/anti-angiogenic factors, hypoxia, and placental insufficiency. Huang et al. postulated that adenosine, a crucial signaling molecule dysregulated in response to hypoxia, contributes to aberrant trophoblast invasion through epigenetic mechanisms involving DNA methylation modifications in specific tissues critical to PE pathophysiology (35). Simultaneously, increased placental DNA methylation of Wnt family member 2 and the metalloproteinase (MMP) promoter region, along with reduced methylation of placental tissue inhibitors of metalloproteinase 3 (TIMP3)—an MMP inhibitor—have been identified in PE. This may account for the loss of normal trophoblast invasion and spiral artery remodeling (36). Moreover, Anderson et al. demonstrated an association between DNA methylation and vitamin D metabolism, suggesting that vitamin D deficiency increases the risk of PE (37).

### 2.2 Histone modification during PE

Histone modifications, including posttranslational modifications such as histone methylation, acetylation, phosphorylation, and ubiquitination, are catalyzed by specific enzymes (38). Acetylation of histones H3 and H4 at specific lysine (K) and arginine (A) residues can modulate gene expression (39). Histone lysine methylation can cause either activation or inhibition, depending on its location (25). Eddy et al. observed a decrease in histone H3 acetylation in response to hypoxia in PE (40). Additionally, regulation of the histone demethylase Jumonji domain-containing 1A and histone deacetylation 2 (HDAC 2) is controlled in response to hypoxia (41). HIF1α is essential for controlling these hypoxia-regulated HDACs, which can exacerbate the PE phenotype by epigenetically altering the DNA packaging protein histone H3 and transactivating target genes (42). Moreover, normal trophoblast development requires interactions between HIF1α and HDAC (43). According to Wang et al., HDAC inhibition correlates with increased expression of chymase, a non-ACE angiotensin-converting enzyme. Chymase has been implicated in inflammation and vascular dysfunction and is upregulated in PE, implying that changes in HDAC expression may contribute to placental dysfunction in PE (44). Syncytin, a key regulator of syncytiotrophoblast (STB) formation, is regulated by the placenta-specific transcription factor and glial cells missing homolog 1 (GCMa). GCMa acetylation is modulated by the cAMP response element-binding protein to activate the cAMP/PKA pathway and induce trophoblast fusion (45). Furthermore, MMP and TIMP expression is regulated by histone H3 Lysine 9/29me3 (46).

### 2.3 ncRNAs

Accounting for 98% of the human genome, ncRNAs do not translate proteins (47) and can be divided into regulatory and housekeeping ncRNAs (48). The former includes miRNAs, long non-coding RNAs (lncRNAs), circular RNAs (circRNAs), piwi-interacting RNAs (piRNAs), small interfering RNAs (siRNAs), transfer RNAs, ribosomal RNAs, and small nuclear RNAs (49). Specifically, regulatory ncRNAs can modulate cellular activity through direct interactions (50). Meanwhile, myriad roles for miRNAs in placental growth have been reported, with their overexpression associated with pregnancy-related illnesses such as PE (51). Notably, differential lncRNA expression has been reported in the placenta and peripheral blood of healthy pregnant women compared to those with PE (52). Despite their implication in various disorders, circRNAs, piRNAs, and siRNAs have been relatively understudied in the context of PE pathophysiology (53).

Gene expression regulation in developing and differentiated tissues is facilitated by several epigenetic mechanisms, including DNA methylation, histone modification, and miRNA activity (21). These mechanisms influence gene expression during placental development and function (54) and may play critical roles in various pregnancy complications such as PE, gestational diabetes mellitus, fetal growth restriction, and preterm birth (55).

## 3 miRNAs and PE

Among ncRNAs, miRNAs—endogenous, short, single-stranded 20–24 nucleotide molecules—were discovered in the early 1990s (56). To date, miRNAs have been detected in nearly all plant and animal species, with more than 2000 identified in the human genome (57). Despite the absence of direct experimental evidence, computational studies have shown that nearly 60% of human genes are possible miRNA targets, suggesting a potential influence on all biological pathways (58). They primarily bind to the 3′ end of messenger RNA (mRNA) molecules and repress target mRNAs through transcript degradation, translation blockage, and gene expression suppression. This implies that miRNAs control target protein gene translation and mRNA degradation at the post-transcriptional level. Consequently, aberrant miRNA expression has been implicated in various malignancies, including ovarian, lung, and breast cancers, by modulating key cellular activities such as cell proliferation, differentiation, apoptosis, angiogenesis, and metabolism (59),.

**Figure 3:**
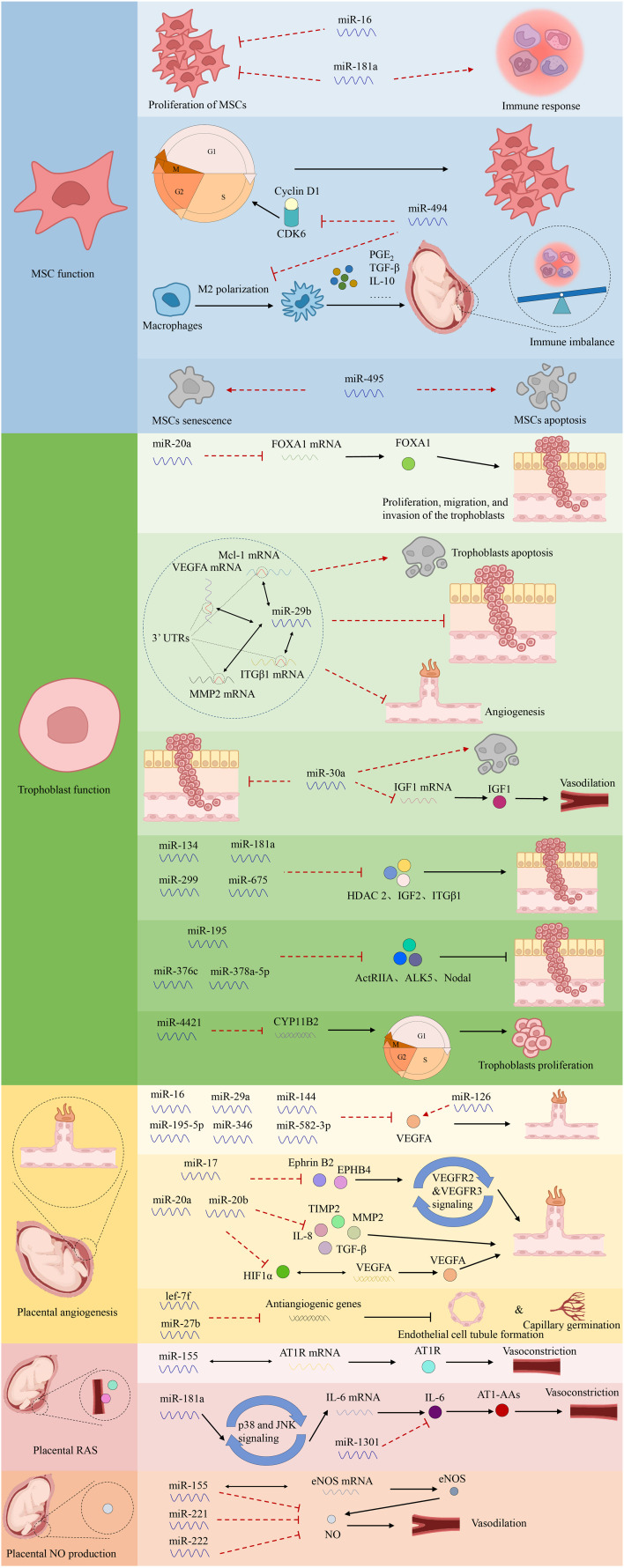
The functions of miRNAs in PE.

Aberrant miRNAs can target downstream genes, leading to decreased trophoblast migration and invasion or enhanced cell death, contributing to PE (24). Certain abnormally expressed miRNAs aggregate in specific chromosomal areas to form closely linked clusters, including the chromosome 14 miRNA cluster (C14MC) and chromosome 19 miRNA cluster (C19MC) (60). C14MC comprises 52 miRNAs that regulate essential physiological processes, including immunological suppression, anti-inflammatory responses, and hypoxia-induced responses (61). Meanwhile, C19MC includes 46 miRNA genes detectable as early as 5 weeks of gestation (62). Overexpression of C19MC is associated with reduced migration in the EVT cell line HTR8/Svneo, suggesting that this cluster mediates decreased trophoblast migration, spiral artery remodeling, and placental ischemia in PE. Conversely, C14MC expression declines as pregnancy progresses (63). Pineles et al. first identified the overexpression of miR-210 and miR-182 in the placenta of patients with PE, representing new targets for PE pathogenesis (15). The first global transcriptome analysis of miRNAs conducted in 2009 using microarray technology revealed that 11 miRNAs were upregulated and 23 were downregulated in women with severe PE compared with controls (64). Differential miRNA expression in PE has also been associated with metabolic changes, transcriptional regulation, immune function, cardiovascular and reproductive development, cell cycle, cell adhesion, and relational signaling pathways, such as transforming growth factor β (TGF-β), Hippo, and mitogen-activated protein kinase signaling pathways, according to biological information analysis (13, 65). However, the roles of miRNAs in the pathophysiology of PE remain to be fully characterized. The investigation of miRNA functions and processes in PE is summarized in **Figure 3**.

### 3.1 Regulation of mesenchymal stem cell function

MSCs in the maternal decidua are central to controlling the pro-angiogenic, immunomodulatory, and anti-inflammatory milieu of the maternal–fetal interface during placentation (66). Alterations in decidual MSC cytokine production and miRNA expression have been observed in PE, with impaired survival, proliferation, and migration of MSCs isolated from women with PE (67, 68). Enhanced miR-181a expression in the umbilical cord and decidual-derived MSCs of patients with PE inhibits proliferation and reduces immunosuppressive qualities without affecting apoptosis (69). Furthermore, increased miR-494 levels in PE decidual MSCs inhibit the G1/S transition, influence proliferation by targeting cyclin D1 and cyclin-dependent kinase 6, and hinder M2 polarization of macrophages. Collectively, this causes an immune imbalance at the maternal–fetal interface mediated by decreased prostaglandin E_2_ secretion (70, 71). Conversely, overexpression of miR-495 accelerates MSC apoptosis and induces cellular senescence (72).

MSCs that show relative low immunogenicity can regenerate damaged tissue through both direct differentiation into tissue cells and indirect support by secreting cytokines to promote the proliferation of tissue cells (73). Furthermore, they can also be induced to adopt immunosuppressive phenotypes to inhibit inflammation and immune activation (74). What’s more, MSCs can facilitate angiogenesis to reconstruct the vascular network and restore blood supply in the placenta (75). MSCs can improve maternal-fetal outcomes in different animal models of PE by enhancing cell metabolism, combating OS, promoting a balance in angiogenesis, and anti-inflammation (66). Studies have demonstrated promising results in the application of MSCs to treat PE, indicating that MSCs could be considered a potential novel treatment option (76). Recent studies indicate that the pleiotropic effects of MSCs arise from the synthesis of soluble paracrine factors, rather than their ability to differentiate (77). Particularly, exosomes alone have been established in studies to be responsible for the therapeutic capabilities of MSCs in PE therapy (78). Mesenchymal stem cell-derived exosomes have the potential to delay the progression of PE and enhance outcomes by improving trophoblast function and placental angiogenesis, regulating immune responses, and reducing inflammation and OS (79).

### 3.2 Regulation of trophoblast function

Abnormally expressed miRNAs primarily regulate trophoblast invasion. Overexpression of miR-20a in PE placentas inhibits proliferation, migration, and invasion of the trophoblast cell line JEG-3 by suppressing forkhead box protein A1 mRNA and protein expression (80). Additionally, up-regulated miR-29b directly binds to the 3′ untranslated regions (UTRs) of myeloid cell leukemia sequence 1, MMP2, VEGFA, and integrin β1 (ITGβ1), inducing trophoblast apoptosis and inhibiting trophoblast invasion and angiogenesis in HTR-8/SVneo and BeWo cells (81). Meanwhile, miR-30a overexpression in JEG-3 cells reduces cell invasion, downregulates insulin-like growth factor 1 (IGF1) mRNA and protein expression, and promotes trophoblast apoptosis in PE (82). Notably, miR-29b and miR-30a are highly expressed in patients with PE (83). Additionally, miR-134, miR-181a, miR-299, and miR-675 inhibit trophoblast invasion and migration by targeting HDAC2, insulin-like growth factor 2, and ITGβ1, with increased circulating and placental levels in PE (84, 85). Conversely, miR-195, miR-376c, or miR-378a-5p promote the proliferation and invasion of HTR8/SVneo cells by targeting the TGF-β pathway components, including activin type II receptor, activin receptor-like kinase 5, and Nodal, with confirmed downregulation in circulating and placental levels in women with PE (84, 86). Gao et al. reported that miR-4421 overexpression in PE downregulates the aldosterone synthase gene (*CYP11B2*), inhibiting trophoblast proliferation and blocking cell cycle progression (83).

### 3.3 Regulation of placental angiogenesis

Several miRNAs may regulate the expression of angiogenesis-related factors in PE (87). Specifically, placental-specific miRNAs may be expressed by human villi trophoblasts, become encapsulated in exosomes, and subsequently released into the maternal circulation. This may partially explain how placental implantation defects contribute to systemic vascular dysfunction (88). Additionally, dysregulated miRNA expression in PE may affect the capillary formation capacity of umbilical vein endothelial cells (70). For example, the expression of miR-16, miR-29a, miR-144, miR-195-5p, miR-346, and miR-582-3p, which target VEGFA—a pro-angiogenic factor promoting vascular endothelial cell proliferation, blood vessel formation, and vascular permeability—is upregulated in PE (89, 90). Moreover, Wang et al. observed decreased viability and proliferative activity of decidua-derived mesenchymal stem cells upon miR-16 overexpression and increased viability in cells transfected with an anti-miR-16 antibody (91). Meanwhile, miR-17, miR-20a, and miR-20b are reportedly overexpressed in PE, potentially cooperating in the suppression of various angiogenesis-related genes, including Ephrin B2 (*EFNB2*), Ephrin type B receptor 4 (*EPHB4*), *HIF1A*, *VEGFA, MMP2, TIMP2*, *TGFB*, and interleukin-8 (*IL8*) (92, 93). In addition, EFNB2 and EPHB4 interact to regulate the internalization and signaling of VEGF receptor 2 (VEGFR2) and VEGFR3, mediating vascular cell adhesion, repulsion, and migration (94, 95). HIF1α, a transcription factor activated in response to hypoxia, regulates the expression of *VEGFA*, emphasizing its importance in placental remodeling during normal pregnancy and its potential role in PE pathogenesis. Additionally, MMP2 and TIMP2 are crucial in spiral artery remodeling during early gestation and in modulating the extracellular matrix during the initial angiogenic response (96, 97). Furthermore, Dicer silencing inhibits lef-7f and miR-27b expression, as well as capillary germination and endothelial cell tubule formation (98). In contrast, the downregulation of miR-126 is associated with pro-angiogenic traits in PE (99, 100). In summary, miR-126 regulates the VEGF pathway at various levels and is inversely related to the angiogenic characteristics of PE (101).

### 3.4 Regulation of placental renin–angiotensin system

Upregulation of anti-angiogenic factors in the placenta, such as soluble fms-like tyrosine-1 (sFlt-1) and soluble endoglin (sEng), contributes to aberrant placental vascularization (102, 103). Increased synthesis of sFlt-1 and endothelin is associated with enhanced development of an agonistic autoantibody against the angiotensin (ANG II) type 1 (AT1) receptor (AT1-AA) in PE (104, 105). Upregulation of AT1-AA correlates with higher blood pressure, elevated endothelin levels, and reduced levels of vasodilators, such as nitric oxide (NO) (106). Pregnant women become insensitive to ANG II-mediated vasoconstriction, maintaining normotension despite elevated renin, aldosterone, and ANG II levels (107). However, sensitivity to ANG II is reportedly enhanced in patients with PE (108). Particularly, the angiotensin II type 1 receptor (AT1R)-specific antibody, AT1-AA, plays a crucial role in PE pathophysiology (109). Notably, elevated levels of AT1-AAs in women with PE are associated with increased production of sFlt-1, sEng, IL-6, and endothelin, increased trophoblast apoptosis, and decreased VEGF expression (110, 111). Specific miRNAs also regulate AT1-AA biosynthesis (25). For instance, miR-155 downregulation correlates with increased AT1R expression (112). Moreover, upregulation of placental miRNA-181a and downregulation of miR-1301 in PE are associated with increased IL-6 production, leading to elevated AT1-AA levels (113, 114).

### 3.5 Regulation of placental NO production

NO, synthesized from L-arginine by nitric oxide synthase (NOS), is a key regulator of vascular resistance and hemodynamic changes during pregnancy (115). Choi et al. reported decreased NO and NOS levels are related to PE (116). In women with PE, the increased expression of miR-155, miR-221, and miR-222 has been linked to decreased NO production (15, 117). Moreover, overexpression of miR-155 in HUVECs is associated with decreased endothelial NOS (eNOS) expression by targeting the 3′ UTR of eNOS (118, 119).

In addition, Yang et al. discovered that miR-148a and miR-152 increase the expression of fatty acid-binding protein 4 in trophoblasts and increase lipid accumulation by regulating DNA methyltransferase 1, which is involved in abnormal lipid metabolism and inflammatory responses in PE pathogenesis (120). Furthermore, miR-1301 is downregulated in PE and may be involved in leptin control throughout pregnancy; however, it is inversely associated with maternal systolic and diastolic blood pressure before delivery (114).

## 4 Roles of exosomes in PE

Extracellular vesicles (EVs)—small semipermeable membrane vesicles—facilitate communication and interaction within or between tissues (121, 122). EVs that exist in myriad bodily fluids are categorized as exosomes, microvesicles, and apoptotic bodies according to the modes of biogenesis and release (123). They are excellent paracrine regulators of cellular crosstalk based on lipid bilayer membranes, DNAs, RNAs, proteins, and lipids (124). In the maternal-placental-fetal unit, microvesicles can be regarded as syncytiotrophoblast membrane microparticles (STBMs). Exosomes (30-150 nm) are microscopic particles formed by the inward folding of the plasma membrane and the creation of intracellular multivesicular bodies (125). STBMs are small vesicles (50-2000 nm) shed from the plasma membrane (126). Apoptotic bodies (500-4000 nm) are produced by apoptotic cells and are characterized by the presence of organelles within the vesicles (127). Exosomes, STBMs, and apoptotic bodies have a similar lipid bilayer membrane and carry genetic and protein cargo associated with the inflammatory reaction in PE (128). However, there are some subtle differences in their roles in PE. Exosomes are believed to have immunosuppressive functions because they can inhibit NK cytotoxicity, suppress T cell activity, and express immunomodulatory proteins such as HLA-G5 (129). Additionally, some studies suggest that exosomes containing miRNAs may have a pro-inflammatory effect by activating various inflammatory pathways in PE (88). Moreover, *in vitro* studies have shown that STBMs can adhere to T cells, B cells, and other immune cells, inducing an inflammatory response in PE (130). Unlike exosomes and STBMs, the target cells of apoptotic bodies are less variable, mainly including macrophages, dendritic cells, and other neighboring cells in PE. Typically in PE, macrophages tend to polarize towards the anti-inflammatory M2 phenotype after phagocytosing apoptotic bodies, and genetic information can be transferred during this process (131).

Particularly, exosomes are released through an endosome-dependent pathway, participate in cell-to-cell communication and are implicated in immune responses, viral pathogenesis, nervous system illness, cancer progression, and pregnancy (132, 133). Exosomes also act as mediators of fetal–maternal communication during implantation and placentation while modulating maternal responses, maintaining cellular metabolic homeostasis, promoting fetal vasculogenesis, and maternal uterine vascular adaptation (134, 135). The total number of circulating exosomes increases throughout gestation, along with pregnancy complications such as gestational diabetes (GDM) and PE (132, 136). The International Society for EVs recommends identifying specific exosome markers, such as CD81, CD9, and CD63 tetraspanins, through techniques like immunoelectron microscopy, flow cytometry, or Western blotting to confirm the exosomal origin of the retrieved vesicles in maternal circulation (137). Pregnancy-associated exosomes have been isolated from the blood of pregnant women at various gestational ages (138). Furthermore, the concentration and content of circulating exosomes may indicate changes in condition, metabolism, fetal growth, and maturation (139). Although the specific origin, payload, and roles of these exosomes in maternal circulation remain unclear, the primary source is the placenta, which releases exosomes into maternal circulation as early as 6 weeks into pregnancy (17). Placental disruption is proposed as a fundamental factor in PE development; therefore, an increased release of exosomes into maternal circulation by placental trophoblasts may be characteristic of this disorder (140).

**Figure 4:**
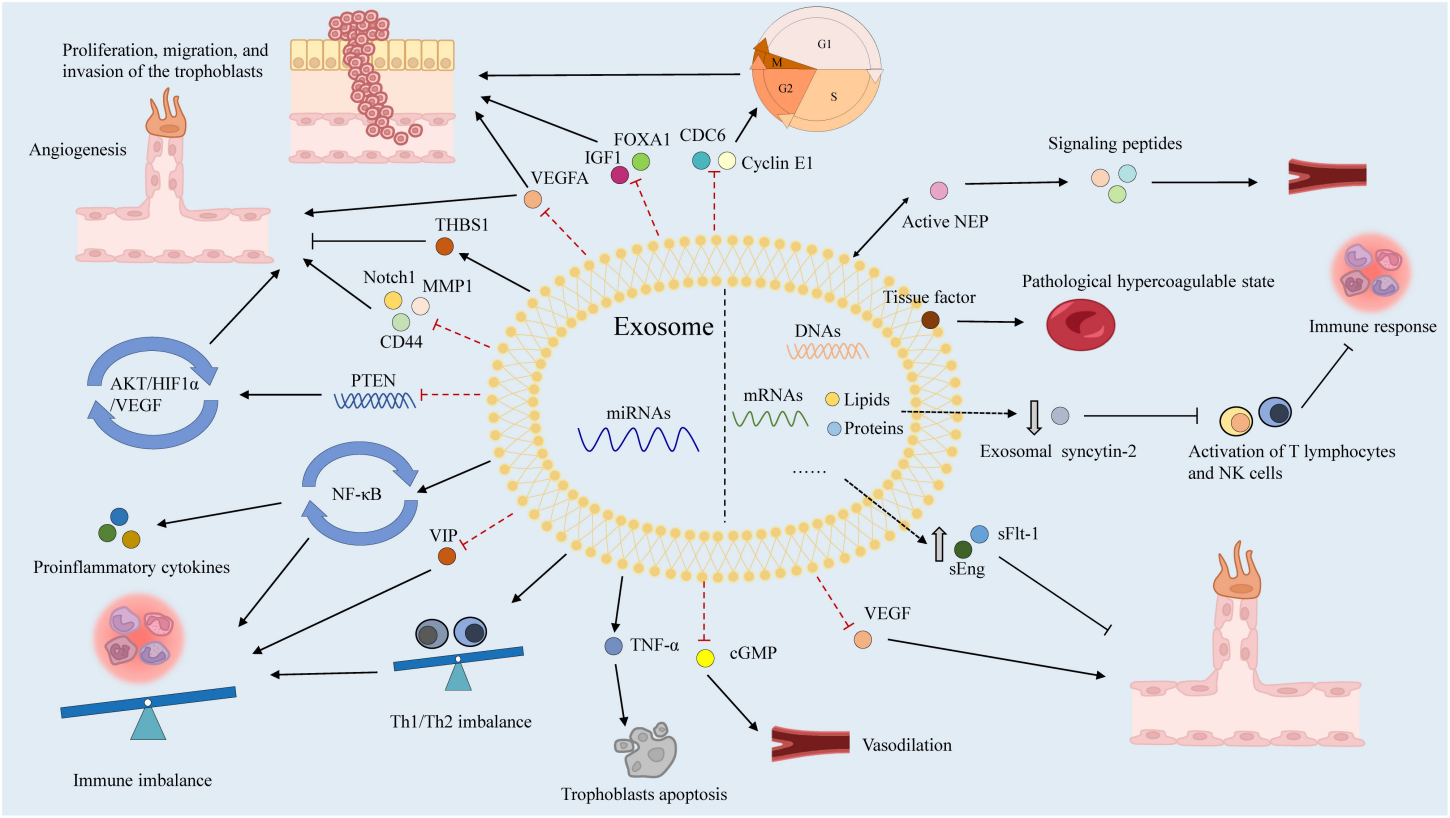
The roles of exosomes in PE.

Numerous humoral factors are involved in PE pathogenesis, characterized by chronic inflammation, leukocyte activation, and elevated blood cytokine levels (141). Increased tumor necrosis factor-α (TNF-α) levels in early pregnancy may induce the expression of intercellular adhesion molecule-1 (ICAM-1) on vascular endothelial cells (ECs) and trophoblasts, activating them within the context of chronic inflammation (142). Notably, soluble ICAM-1 can be released by leukocytes adhering to the vascular endothelium due to OS (143). Additionally, chronic inflammation activates lymphocyte function-associated antigen-1 in leukocytes, disrupting spiral artery remodeling with EC and trophoblast activation (144). These two EC activation pathways cause vascular dysfunction, contributing to PE. In these patients, immune tolerance to trophoblasts is maintained by the impaired interaction between human leukocyte antigen-G and decidual natural killer (NK) cells, dendritic cells, regulatory T (Treg) cells, and cytokines secreted by uterine NK cells in the decidua, resulting in EC and trophoblast dysfunction (145, 146). Subsequently, EVTs fail to adequately invade the uterine decidua and myometrium, resulting in impaired spiral artery remodeling. Type-1 T helper (Th1) cells and Th17 cells, which secrete proinflammatory cytokines including TNF-α, interferon-γ, IL-6, and IL-17, are also prominent in PE (140). Disruption of angiogenesis causes vascular dysfunction, contributing to poor placentation and PE pathogenesis (147). Increased levels of antivascular growth factors, including sFlt-1 and sEng, can decrease placental growth factor (PlGF) levels and reduce angiogenesis in the placenta (148). Exosomes reportedly transport various humoral factors, including miRNAs, to distant organs and may have important roles in PE (**Figure 4**).

### 4.1 ExomiRs in PE

Exosomes that contain miRNAs, known as exomiRs, can be absorbed by nearby or distant cells and are modified by receivers (149). In addition to their complex with argonaute (AGO) proteins, miRNAs are actively released by bundling in exosomes to avoid destruction by RNases (150, 151). Compared to AGO-bound miRNAs, a small portion of the total miRNA plasma population is made up of exomiRs, which are required for donor–receiver cell interactions and impact functional outcomes by modifying host gene expression in recipient cells (152, 153). As exomiRs are specifically packaged into exosomes and released by the cell to initiate cellular reprogramming, they have recently become important biomarkers for several illnesses (154, 155).

Placenta-derived exosomes contain abundant miRNAs that are regulated by various factors such as hypoxia, environmental factors, and epigenetic alterations (156). There are more than 600 exomiRs expressed in the placenta, which play critical roles in placental development and function by regulating the expression of genes involved in trophoblast proliferation, differentiation, invasion, migration, apoptosis, and angiogenesis (157). ExomiRs expression patterns in the placenta shift during gestation, for example, a comparison of placentas isolated from the first and third trimesters revealed a total of 208 miRNA transcripts that were differently expressed (158). In particular, exomiRs that regulate trophoblast proliferation and angiogenesis are significantly expressed in the first trimester, while exomiRs that promote trophoblast differentiation and reduce trophoblast proliferation are strongly expressed in the third trimester (158). Considering the upregulation of miRNAs in hypoxic cells and their long half-life and high stability, the relationships between miRNAs, including exomiRs, and PE have been extensively studied (159, 160). Different concentrations of exomiRs may be associated with the pathological state of PE (**Table 1**). Devor et al. demonstrated that women with PE and those with normal pregnancies exhibit different exomiR patterns during the first trimester, which may be exploited for early PE diagnosis (140). Moreover, exosomes secreted by cytotrophoblasts, which express placenta-specific miRNAs, participate in embryo implantation by promoting Treg cell differentiation and suppressing the nuclear factor-B signaling pathway, influencing the immune reaction and inflammatory response at the fetal–maternal interface and remodeling the spiral artery (122). ExomiRs, such as miR-517a, released from trophoblasts into the maternal circulation also have an important role in regulating the Th1/Th2 balance, leading to immune response activation, and their imbalance may contribute to PE pathogenesis (18). Notably, enrichment of specific exomiRs causes aberrant cell proliferation, adhesion, migration, and invasion in women with PE (177). Placental-specific miRNAs released into the maternal circulation by placental trophoblasts in exosomes may also impact TNF signaling regulation, which can induce vascular activation and dysfunction in placental trophoblasts by activating leukocytes and inducing vascular endothelial adhesion molecules (88). Moreover, the implanted embryo releases exomiRs to control blood flow (178).

**Table 1:** A summary of exomiRs related to PE.

ExomiRs	Mechanism	Reference
ExomiR-16	In PE patients, overexpressed exomiR-16 is negatively correlated with the levels of VEGFA that influences proliferation and migration of trophoblasts and angiogenesis processes, moreover, overexpressed exomiR-16 induces cell-cycle arrest by targeting cyclin E1.	(91, 161)
ExomiR-17-5P	ExomiR-17-5p could target PTEN to activate AKT/HIF1α/VEGF signaling pathway related to angiogenesis such as migration and tube formation of ECs in PE.	(161, 162)
ExomiR-122-5p	Upregulated exomiR-122-5p of PE patients negatively regulates ECs migration.	(163)
ExomiR-126-5P	ExomiR-126–5p mediates pro-angiogenic effects through inhibition of THBS1 expression, and enhanced proliferation and migration of ECs.	(161, 164)
ExomiR-150	ExomiR-150 stimulates the proliferation and migration of ECs, and expresses higher VEGF and Notch1, indicating a pro-angiogenic impact.	(122, 165)
ExomiR-151a-5p	Upregulation of exomiR-151a-5p is associated with poor proliferation and migration of ECs in PE.	(163)
ExomiR-153-3p	ExomiR-153-3p overexpression reduces cell proliferation and invasion while increasing apoptosis. Furthermore, it can bind the 3’UTRs of HIF1α mRNA and limit its expression, which has been linked to decreased tube formation in primary human umbilical vein ECs, decreased VEGFA production and angiogenesis.	(8, 166)
ExomiR-210-3p	Downregulation of exomiR-210-3p markedly decreased the tube formation, migration and proliferative capacities of ECs in PE.	(163)
ExomiR-215-5p	ExomiR-215-5p overexpression in pregnant women reduces trophoblast proliferation and migration during PE via reducing CDC6, a necessary gene code for DNA replication. Furthermore, it also inhibits the EMT via reducing CDC6 expression through epigenetic downregulation of E-cadherin.	(167)
ExomiR-296-5p	Overexpression of exomiR-296-5p in PE results in downregulation of PTEN and upregulation of PTEN/PI3K/AKT signaling pathway related to angiogenesis such as migration and tube formation of ECs.	(163, 168)
ExomiR-342-3p	ExomiR-342-3p inhibits the proliferation and migration of ECs and results their dysfunction.	(169, 170)
ExomiR-376c-3p	Upregulated exomiR-376c-3p negatively regulates ECs migration in PE.	(163)
ExomiR-486-1-5p	ExomiR-486-1-5p participates in angiogenesis, migration and placental development by targeting IGF1.	(132)
ExomiR-486-2-5p	ExomiR-486-2-5p participates in angiogenesis, migration and placental development by targeting IGF1.	(132)
ExomiR-499	ExomiR-499 suppresses proinflammatory cytokine production by decreasing NF-κB signaling, reducing inflammatory responses and generating an immune-tolerant microenvironment in the uterus.	(171)
ExomiR-517a	ExomiR-517a is crucial for controlling the Th1/Th2 balance, which triggers the immune system. In this particular case, the imbalance may be the cause of PE development.	(18)
ExomiR-517b	ExomiR-517b can increase the expression of TNF-α and/or other death ligands.	(88)
ExomiR-520c-3p	Upregulation of exomiR-520c-3p inhibits EVTs invasion by targeting CD44 in PE.	(172)
ExomiR-525-5p	Overexpression of exomiR-525-5p can decrease the anti-inflammatory factor VIP that may participate in the occurrence of PE.	(173)
ExomiR-526b-5p	ExomiR-526b-5p modulates MMP1 and HIF1α expression that are important in the remodeling of the spiral arteries.	(174)
ExomiR-550a-5p	ExomiR-550a-5p positively regulates cell migration.	(163)
ExomiR-571a-3p	ExomiR-571a-3p represses cGMP-dependent protein kinase 1 by NK cells, a key mediator of nitric oxide signaling.	(175)
ExomiR-1269b	ExomiR-1269b regulates the expression of the FOXO1, which is critical in endometrial stromal decidualization and implantation.	(176)

ECs, endothelial cells; EMT, epithelial-mesenchymal transition; exomiRs, exosomal microRNAs; EVTs, extravillous trophoblasts; FOXO1, forkhead box O1 gene; HIF1α, hypoxia-inducible factor 1-α; IGF1, insulin-like growth factor 1; MMP, metalloproteinase; PE, preeclampsia; PTEN, phosphatase and tensin homolog; THBS1, thrombospondin-1; TNF-α, tumor necrosis factor-α; UCMSCs, umbilical cord mesenchymal stem cells; UTRs, untranslated regions; VEGFA, vascular endothelial growth factor A; VIP, vasoactive intestinal peptide.

Lower expression of miR23a-3p, miR-125b-2-3p, miR-144-3p, miR-192-5p, miR-205-5p, miR-208a-3p, miR-335-5p, miR-451a, miR-518a-3p, and miR-542-3p has been reported in exosomes isolated from patients with PE compared to normal controls, whereas the expression of let-7a-5p, miR-17-5p, miR-26a-5p, miR-30c-5p, miR-141-3p, miR-199a-3p, miR-210, miR-221-3p, miR-325-3p, miR-516-5p, miR-517, miR-520a, miR-525, miR-526a, miR-584-5p, miR-7445p, and miR-6724-5p is higher in patients with PE (121, 179). In addition to these exomiRs, miR-150 is a significant regulator of angiogenesis *in utero* that increases the expression of VEGF and Notch1. miR-150 expression is upregulated in umbilical cord mesenchymal stem cell-derived exosomes from a healthy pregnancy in piglets (165). Meanwhile, miR-153-3p is upregulated two-fold in exosomes from patients with PE. Its overexpression decreases cell proliferation and invasion while promoting apoptosis (8). Furthermore, miR-153-3p can bind the 3′ UTRs of *HIF1A* mRNA and inhibit its expression, which is associated with decreased tube formation by primary human umbilical vein ECs, decreased VEGFA production, and reduced angiogenesis (166, 169). Overexpression of exomiR-215-5p in PE reduces trophoblast proliferation and migration by decreasing cell division cycle 6 expression via the epigenetic downregulation of E-cadherin, which is required for DNA replication (167). Placental miR-342-3p, isolated from PE exosomes, has been implicated in EC dysfunction (180). ExomiRs, such as miR-486-1-5p and miR486-2-5p, participate in angiogenesis, migration, and placental development by targeting IGF1 (181). In contrast, hsa-miR-525-5p, hsa-miR-526b-5p, and hsa-miR-1269b are expressed exclusively under diseased conditions (182). Notably, hsa-miR-525-5p can decrease the expression of the anti-inflammatory factor vasoactive intestinal peptide (173). Meanwhile, hsa-miR-526b-5p modulates MMP1 and HIF1α expression (174), and hsa-miR-1269b regulates the expression of the forkhead box O1 gene (*FOXO1*), which is important for endometrial stromal decidualization and implantation (176).

### 4.2 Other functions of exosomes in PE

STB microvilli produced in the placenta may impede the proliferation and growth of ECs and serve as pathophysiological markers of PE (183). High levels of exosomes are released into the maternal circulation by STBs, causing endothelial dysfunction and leading to vascular constriction in PE (184). Exosomes may supply proteins to trophoblasts, generating a supportive environment that interferes with the operation of distant organs (18). Neprilysin (NEP), which may participate in PE pathogenesis by activating signaling peptides, such as endothelin and atrial natriuretic peptide, is widely expressed in placental trophoblasts and may directly promote impaired uteroplacental circulation (185, 186). Active NEP is reportedly secreted into the maternal circulation and coupled to STB-derived exosomes. Its expression is enhanced in PE, indicating that STB-derived exosomes could act as a bridge between placental dysfunction and subsequent clinical maternal disorders associated with PE (187). Additionally, various tissue factors are expressed on the surface of STB-derived exosomes, and its overexpression is related to PE (188). Moreover, exosomal syncytin-2 levels are significantly lower in the blood of patients with PE, which may be associated with STB production by villous trophoblasts (189). Furthermore, PE-derived exosomes participate in vascular dysfunction via their overexpression of sFlt-1 and sEng, which inhibit EC proliferation, migration, and differentiation, resulting in endothelial dysfunction (190). Notably, exosomes from PE may contribute to the spread of endothelial injury by sequestering free VEGF in maternal circulation (191).

## 5 Biomarker functions of exomiRs

Remarkably, the transfer of miRNAs by exosomes may result in the transmission of genetic information and generation of diverse proteins. ExomiRs are responsible for various physiological and pathological conditions in target cells and have been extensively investigated as biomarkers for the diagnosis, prediction, and treatment of many diseases, such as GDM and breast cancer (122).

**Figure 5:**
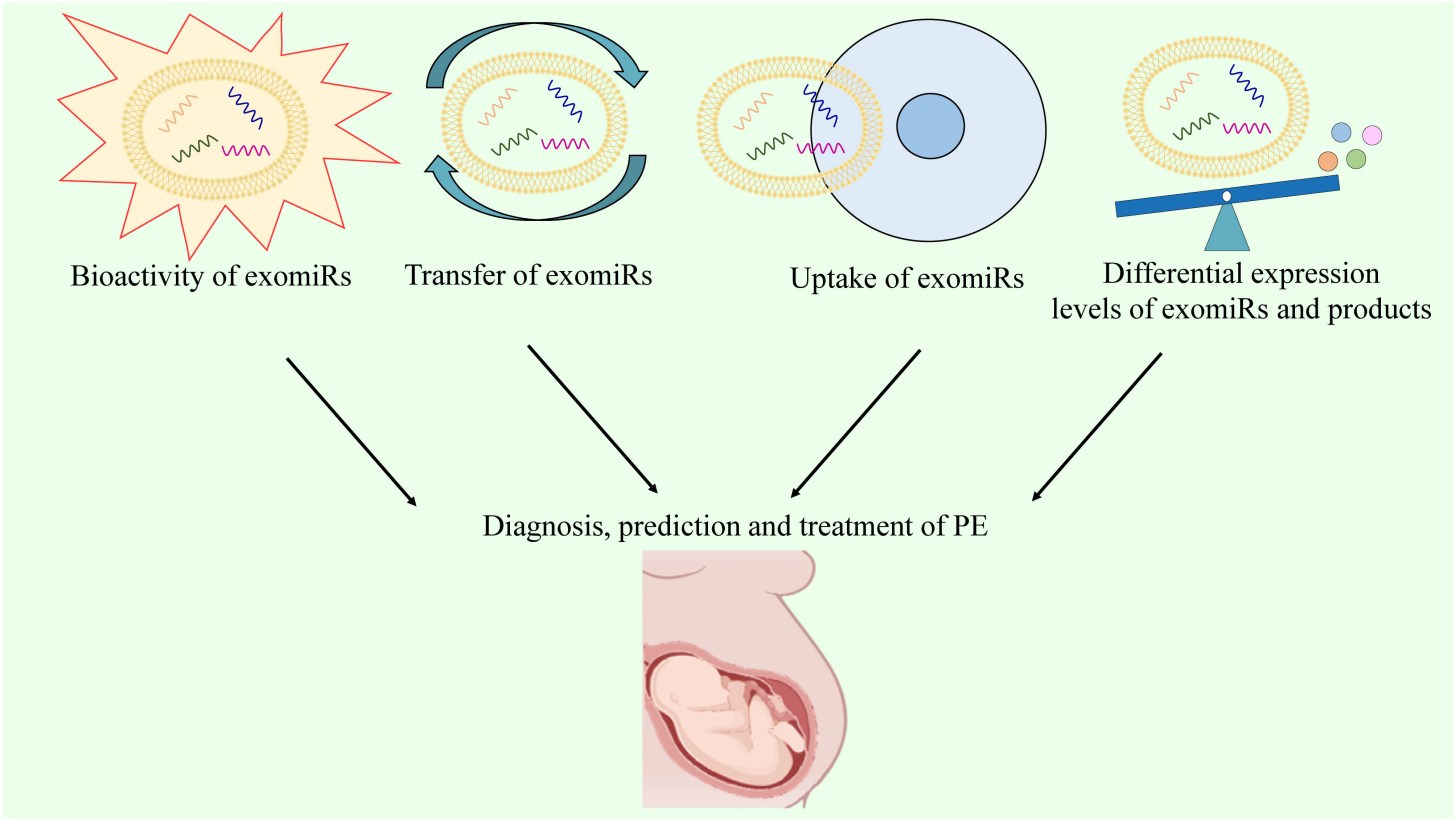
The prospects of exomiRs in PE.

Although multiple biomarkers, including sFlt-1, sEng, and PlGF, have been developed for PE diagnosis, their sensitivity and specificity are suboptimal due to the interference of many molecules. A reliable serum biomarker is required for an accurate and early diagnosis of PE. Molecular biomarkers, rather than biochemical indicators, may provide a more reliable platform for screening and diagnosing PE. The bioactivity, transfer, and, most importantly, uptake of exomiRs by target cells have been reported in pathological conditions associated with PE (192). For PE, exomiRs can be regarded as diagnostic biomarkers and therapeutic targets based on their differential expression levels (18, 122). Hence, detecting the expression of exomiRs and their gene products, such as exomiR-210-3p, may be useful for the diagnosis and prediction of PE. The ability of exosomes to transport vital information across cells can be exploited to improve clinical efficiency by leveraging their ability as biomarker candidates and prominent treatment targets (**Figure 5**). Recently, immune abnormalities in the placenta and maternal circulation have been found to occur before the clinical onset of PE (193). Particularly, Excessive systemic and placental complement activation, as well as poor adaptive T cell tolerance with Th1/Th2/Th17/Treg imbalance, have been observed in both human and animal models of PE (194). This indicates that the potential of immune-modifying therapy for preventing or treating PE is significant, despite the limited existing evidence (195). As mentioned above, exomiRs can regulate the Th1/Th2 balance and immune response, indicating that the regulation of exomiRs may help treat PE. Particularly, several studies have identified exomiR-15a-5p as a potential therapeutic target for PE due to its ability to inhibit trophoblast viability, migration, invasion, and epithelial-to-mesenchymal transition in trophoblasts (196).

Currently, there are no effective pharmaceutical treatments or strategies to prevent PE. Traditionally, exercise, low-dose aspirin and calcium intake are recommended to prevent PE in high-risk women by professional societies (197). Nowadays, statins, etanercept, sulfasalazine, hydroxychloroquine, eculizumab, metformin, and proton pump inhibitors are gaining more and more attention from researchers (9, 198). This review may provide a new idea for the prevention of PE. Targeting exomiRs and their related signaling pathways, transcripts, etc., may play a role in the prevention of PE. Research on PE is ongoing and will lead to innovative preventive methods as our knowledge of the condition progresses.

## 6 Conclusions and perspectives

The diagnosis, prediction, and treatment of PE are challenging owing to its complicated and heterogeneous nature. With the advancement of epigenetic research, distinct layers of the human placenta have been found to express hundreds of miRNAs associated with cell proliferation, differentiation, and apoptosis, implying an association between aberrant miRNA expression and PE. Specific miRNAs can influence MSC function by regulating their survival, proliferation, and migration. Moreover, several miRNAs are involved in the proliferation, migration, and invasion of trophoblasts, regulating their functions. In addition to controlling the expression of different factors related to angiogenesis, such as VEGFA, MMP2, and TIMP2, miRNAs contribute to the regulation of the placental renin–angiotensin system by controlling the expression of anti-angiogenic factors, such as sFlt-1 and sEng, associated with vascular resistance. Moreover, certain miRNAs regulate placental NO production and are linked to maternal blood pressure. In PE, exosomes may contain molecules with characteristics of damaged trophoblasts, including miRNAs and other proteins such as NEP and VEGF. The exosomal lipid bilayer is protected from the extracellular environment, making it an ideal delivery mechanism for miRNAs and endowing exomiRs with improved stability and protection from degradation compared with miRNAs.

In particular, exomiRs have the characteristics of disease-causing cells, and considering their significance and stability, they may represent an effective non-invasive method for the early diagnosis of PE in maternal peripheral blood. Additionally, exosomes in PE may include miRNAs with the characteristics of injured trophoblasts. Accordingly, analyzing exomiRs may aid in predicting the onset of PE. Moreover, exomiRs may be viable targets for treating PE. Since exomiRs can lead to the occurrence and development of PE through different pathways, inhibiting the expression of these exomiRs and blocking these pathways may be effective for treating PE. Specifically, measuring exomiRs, such as exomiR-210-3p and exomiR-520c-3p, may improve our ability to identify patients with PE earlier. Furthermore, varying levels of exomiR expression may be associated with the development and prognosis of PE, increasing the accuracy of predicting PE progression and clinical outcomes. That is, determining the concentration of exomiRs may assist in predicting the progression and prognosis of PE. Considering the overexpression and downregulation of exomiRs and their expression products, future PE treatments may involve targeting these exomiRs, specific signaling pathways, and downstream gene products, such as the PTEN/PI3K/AKT signaling pathway and IGF1.

In conclusion, the pathogenesis of PE has not been fully elucidated, and its early diagnosis, prevention, and treatment are significant but currently limited. The ability to identify exomiRs generated during pregnancy and implement non-invasive therapies to mitigate their effects may prove critical in clinical applications for the diagnosis, prediction, and treatment of PE. For example, it may be helpful for the diagnosis and prediction of PE by detecting the expression levels of exomiRs and transcripts. Additionally, targeting exomiRs and related pathways may have great potential for the treatment of PE. However, further research is warranted to identify the mechanisms underlying the release of exomiRs involved in PE progression.

## Author contributions

YS: Writing – original draft. BH: Writing – original draft. JW: Writing – original draft. AC: Writing – review & editing. SL: Writing – review & editing.
